# Aging-Induced Reduction in Safflower Seed Germination via Impaired Energy Metabolism and Genetic Integrity Is Partially Restored by Sucrose and DA-6 Treatment

**DOI:** 10.3390/plants13050659

**Published:** 2024-02-27

**Authors:** Tang Lv, Juan Li, Lanyu Zhou, Tao Zhou, Hugh W. Pritchard, Chaoxiang Ren, Jiang Chen, Jie Yan, Jin Pei

**Affiliations:** 1 State Key Laboratory of Southwestern Chinese Medicine Resources, Chengdu University of Traditional Chinese Medicine, Chengdu 611137, China; tanglv2024@163.com (T.L.); juanli@stu.cdutcm.edu.cn (J.L.); 2020ks360@stu.cdutcm.edu.cn (L.Z.); zhoutao@cdutcm.edu.cn (T.Z.); chaoxiangren@cdutcm.edu.cn (C.R.); janshen1986@163.com (J.C.); 2 College of Pharmacy, Chengdu University of Traditional Chinese Medicine, Chengdu 611137, China; 3 Kunming Institute of Botany, Chinese Academy of Sciences, 132 Lanhei Road, Heilongtan, Kunming 650201, China; hwp@mail.kib.ac.cn; 4 Royal Botanic Gardens, Kew, Wakehurst, Ardingly, Haywards Heath RH17 6TN, West Sussex, UK

**Keywords:** controlled deterioration treatment (CDT), DA-6, differentially expressed genes, seed aging, seed germination recovery, sucrose

## Abstract

Seed storage underpins global agriculture and the seed trade and revealing the mechanisms of seed aging is essential for enhancing seed longevity management. Safflower is a multipurpose oil crop, rich in unsaturated fatty acids that are at high risk of peroxidation as a contributory factor to seed aging. However, the molecular mechanisms responsible for safflower seed viability loss are not yet elucidated. We used controlled deterioration (CDT) conditions of 60% relative humidity and 50 °C to reduce germination in freshly harvested safflower seeds and analyzed aged seeds using biochemical and molecular techniques. While seed malondialdehyde (MDA) and fatty acid content increased significantly during CDT, catalase activity and soluble sugar content decreased. KEGG analysis of gene function and qPCR validation indicated that aging severely impaired several key functional and biosynthetic pathways including glycolysis, fatty acid metabolism, antioxidant activity, and DNA replication and repair. Furthermore, exogenous sucrose and diethyl aminoethyl hexanoate (DA-6) treatment partially promoted germination in aged seeds, further demonstrating the vital role of impaired sugar and fatty acid metabolism during the aging and recovery processes. We concluded that energy metabolism and genetic integrity are impaired during aging, which contributes to the loss of seed vigor. Such energy metabolic pathways as glycolysis, fatty acid degradation, and the tricarboxylic acid cycle (TCA) are impaired, especially fatty acids produced by the hydrolysis of triacylglycerols during aging, as they are not efficiently converted to sucrose via the glyoxylate cycle to provide energy supply for safflower seed germination and seedling growth. At the same time, the reduced capacity for nucleotide synthesis capacity and the deterioration of DNA repair ability further aggravate the damage to DNA, reducing seed vitality.

## 1. Introduction

Seed physiological quality is a complex trait that includes aging tolerance, germination efficiency, and seedling establishment [1]. Thus, high-quality seeds are strongly associated with strong field emergence and productivity. Seed physiological quality is regulated by the complex interactions between endogenous genetic control factors and external environmental factors during seed set on the parent plant [2]. Intrinsic factors include species-specific seed longevity characteristics and chemical composition. High temperature and humidity are the main external factors affecting the relative rate of seed viability loss and associated decline in seed vigor [3,4]. The process of seed viability loss includes altered cell metabolism and reduced genetic integrity and DNA repair mechanisms. For instance, the degradation of oil bodies and membrane lipids [phosphatidylcholines (PC), phosphatidylethanolamines (PE), phosphatidylserines (PS), phosphatidylinositols (PI), Phosphatidylglycerols (PG)], and ultimately the destruction of the cell membrane structure, is associated with decreased seed viability [5]. Also, seed aging severely impairs key pathways of carbohydrate metabolism, lipid metabolism, and antioxidant activity, e.g., in Siberian wild rye [6].

Thus, seeds contain macromolecules and nutrients such as lipids, starch, and proteins [7], that are all susceptible to degradation during seed aging. These macromolecules are attached by the tricarboxylic acid cycle (TCA), which is the primary energy-producing pathway in seeds. During the imbibition process of oil seeds, triacylglycerols (TAG) are hydrolyzed by lipases to release glycerol and FAs. FAs support seed germination by generating CoA-SH via the β-oxidation pathway, which leads to the formation of oxaloacetate via the TCA or the glyoxylate cycle. Finally, gluconeogenesis enables the reverse conversion of glycolipids to sucrose [8,9]. Glycerol, another product of TAG hydrolysis, is converted by glycerol-3-phosphate dehydrogenase into glycerol-3-phosphate, which is then further converted into dihydroxyacetone phosphate (DHAP). DHAP is involved in the glycolysis pathway and is utilized during seed germination. Soluble sugars are eventually catabolized into ATP which is the most direct form of energy for seed germination and growth [10]. However, in aging soybean and sunflower seeds, fatty acids’ conversion into sucrose can be hampered at the early stage of germination and is recognized as the principal cause for decreased soybean seed vigor after storage [11,12]. *Sugar-dependent 1* (*SDP1*) is the enzyme responsible for the initial step of TAG hydrolysis. In Arabidopsis, the hydrolysis of triacylglycerol in *SDP1* seeds is blocked, leading to a stunted growth phenotype even in the absence of sucrose, which mimics the phenotype of *SDP6* [13]. The conversion of glycerol and fatty acids to sucrose was significantly impaired in *SDP6* seeds, and seedlings showed a marked growth arrest phenotype, which was rescued by exogenous sucrose [14]. Seed germination and other physiological activities rely on the supply of energy, especially before seedlings obtain photosynthetic capacity. Hence, alterations in energy substances during seed aging are potentially hugely influential on seed physiological quality.

Plant cells and tissues face constant challenges to the stability and integrity of their genetic material. These challenges come from both internal and external factors that can cause DNA damage [15]. Reactive oxygen species (ROS), UV radiation, ionizing radiation, and other factors can cause DNA damage. When damaged DNA is not promptly repaired, essential biological processes such as DNA replication, recombination, and transcription may fail, leading to cell death [16]. Thus, aged rice and soybean seeds have increased frequency of embryonic DNA damage and chromosomal alterations in root cells [17]. DNA damage during imbibition can also delay the germination process [18]. Maintenance of genome integrity depends on its reliable replication in proliferating cells and its efficient repair in all cell types [19]. In plants, DNA strand breaks are repaired by the homologous recombination (HR) and non-homologous end joining (NHEJ) pathways, which play a vital role in double-strand break repair [20]. Proliferating cell nuclear antigen (PCNA) is a pivotal protein that participates in multiple DNA repair pathways, including homologous recombination (HR), non-homologous end-joining (NHEJ), base excision repair (BER), mismatch repair (MMR), and nucleotide excision repair (NER). KU70/80 heterodimer is a pivotal protein in core-NHEJ machinery and recruits other proteins to facilitate DNA break repair [21]. Moreover, oxidative DNA damage induced by ROS is thought to be a major source of genomic instability and senescence [22]. Seeds can decrease DNA damage by scavenging ROS through antioxidant enzymes, including CAT, and other antioxidant mechanisms. Additionally, RNA integrity decline has been associated with a decrease in germination potential in soybeans during prolonged storage [23].

Safflower (*Carthamus tinctorius* L.) is a versatile economic crop that is cultivated worldwide for various purposes including as a dye source, oilseed livestock feed, and for medicinal purposes [24]. Its seeds are used to produce edible oil due to the enriched unsaturated fatty acids (oleic acid and linoleic acid) and lipid concomitants [25]. The seed oil content of safflower averages 40% of its dry weight (DW), especially linoleic acid, which is ranked number one among the kingdom of oilseed crops [26]. However, being rich in unsaturated fatty acids makes safflower seeds susceptible to oxidation, leading to seed deterioration during storage, and reduced germination and seedling establishment [27,28]. For example, after being stored under ambient conditions for just 14 months, the safflower seed germination rate decreases dramatically, and viability is lost [29]. Therefore, further research is required to investigate the specific causes of germination deterioration and to explore effective mitigation approaches, since safflower seeds are susceptible to aging.

Previous studies have shown that changes in sugar, lipids, and plasma membrane structure play a decisive role in the different stages of safflower seed aging [30]. However, molecular mechanisms of energy substance metabolism and genetic material damage have not yet been explored. To explore this question, we propose a hypothesis based on previous research: impaired metabolic pathways of energy substances and disruption of the integrity and stability of the genetic material play a deterministic function in the aging process of safflower seeds. We used CDT to rapidly age seeds and observed changes in energy reserve depletion and DNA replication and repair genes during aging. A large number of differentially expressed genes (DEGs) were identified during the process of safflower seed aging containing starch and sucrose metabolism, fatty acid metabolism, tricarboxylic acid cycle, DNA replication, and DNA repair pathways. Finally, we treated aged safflower seeds with exogenous sucrose and DA-6, and seed viability was improved. This study will elucidate the key molecular mechanisms and regulatory networks involved in safflower seed aging, laying a foundation for preserving safflower germplasm resources.

## 2. Results

### 2.1. CDT Significantly Decreases Seed Germination and Seedling Establishment

**Figure 1:**
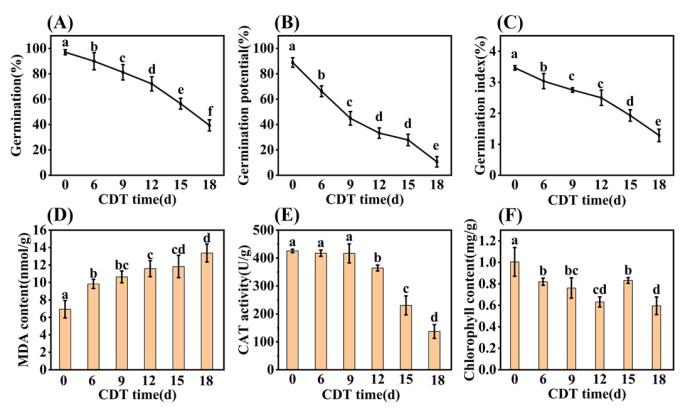
Effect of CDT over 18 days on seed germination level and biochemical properties. (**A**) Final germination; (**B**) germination potential; (**C**) germination index; (**D**) malondialdehyde (MDA) content; (**E**) catalase (CAT) activity; and (**F**) chlorophyll content of the seedling leaf. The seed germination data were obtained seven days after germination. Data are mean ± SE. Different lowercase letters indicate significant differences at *p* < 0.05, with three biological replicates for all treatments.

The germination and seedling establishment of safflower seeds decreased significantly with the increase in CDT time (Figure 1). At the beginning of aging, the germination of C0 (day 0) and C6 (day 6) were determined as 97% and 90%, respectively. After C9 (day 9) treatment germination was 79%, and significantly lower than that of unaged (C0) seeds (Figure 1A). As aging proceeded through C12 (day 12) and C15 (day 15), germination was 72% and 56%, respectively. By C18 (day 18) germination was only 39%. Seed germination potential and germination indexes also fell with CDT treatment, concomitant with decreasing germination (Figure 1B,C).

Biochemical changes during CDT were also determined. The level of malondialdehyde (MDA) content showed a significant increase under CDT conditions (Figure 1D). Compared with unaged seeds, the MDA level increased by 42%, 53%, 67%, 71%, and 93% for C6, C9, C12, C15, and C18 aging times, respectively (Figure 1D). Additionally, catalase (CAT) activity significantly decreased with aging from C9, reaching 70% and 38% of that in unaged seeds at C15 and C18, respectively (Figure 1E).

**Figure 2:**
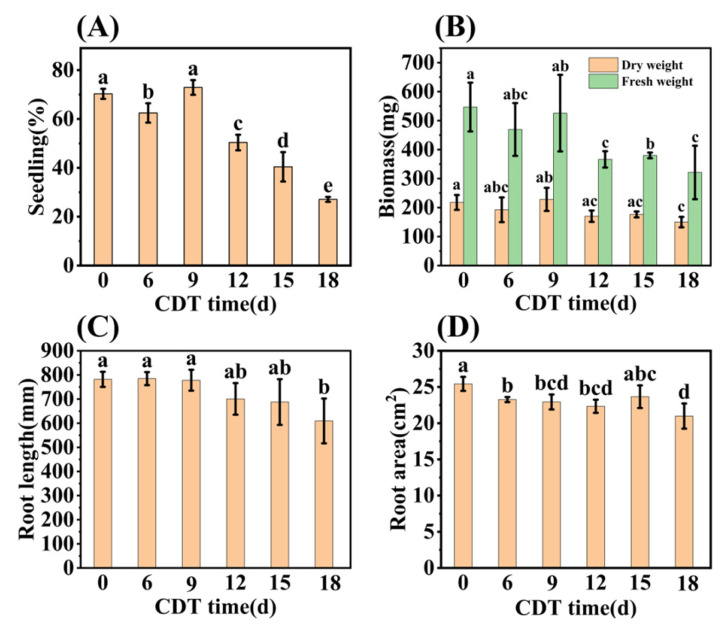
CDT of safflower seeds reduces their seedling establishment. (**A**) Seedling establishment rate of C0, C6, C9, C12, C15, and C18 (days) aged seeds (3 weeks after sowing); (**B**) seedling biomass; (**C**) root length; and (**D**) root surface area of seedlings. The seedling data are obtained 3 weeks after sowing. Data are mean ± SE. Different lowercase letters indicate significant differences among treatments (*p* < 0.05).

Early seedling establishment was also investigated during aging and found to completely decrease with increased CDT times (Figure 2A and Figure S1). Specifically, Figure 2B shows that the seedling fresh weight (FW) and dry weight (DW) fell about 40% and 30% as aging progressed from C0 (546.90 mg and 217.69 mg, respectively) to C18 (321.23 mg and 149.91 mg, respectively). Meanwhile, the root system analysis revealed that the root length and root surface area decreased with an increase in aging days (Figure 2C,D). Moreover, the chlorophyll content of seedlings trended downwards during aging (Figure 1F). The chlorophyll content of safflower seeds for C0 and C9 were 1.00 and 0.82 mg·g^−1^, respectively, and only 0.60 mg·g^−1^ for C18 seeds (Figure 1F). Taken together, these results corroborate the previous conclusion that the CDT process significantly reduces the ability of safflower seeds to germinate and the seedlings’ establishment.

### 2.2. Expression of Genes and Kyoto Encyclopedia of Genes and Genomes (KEGG) Analysis of Energy Metabolism during Seed Aging

**Figure 3:**
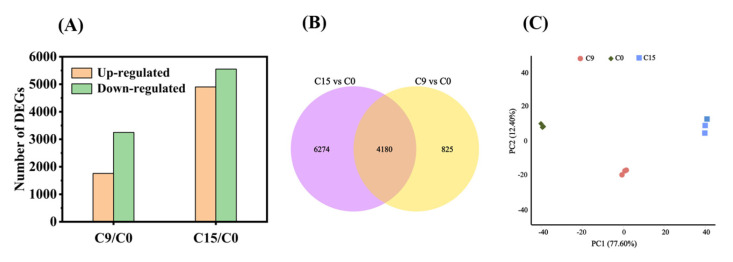
The profile of the differentially expressed genes. (**A**) Number of DEGs identified different comparison groups with different CDT time; (**B**) Venn diagram illustrating the number of identified DEGs in the two different comparison groups (C9/C0, C15/C0); and (**C**) PCA of C0, C9, C15. Each treatment had three biological repeats.

Previous studies have shown that a germination level of 85% is a critical node (CN) for safflower seeds [30]. In this experiment, we observed that the germination level of C9 seeds was concentrated near the CN, whereas for C15, only approximately half of the seed population survived. Therefore, we selected C9 and C15 seeds for transcriptome sequencing. We classified the DEGs into two groups based on the time intervals of aging: C9/C0 and C15/C0. A total of 15,459 genes (Log_2_ (Fold change) ≥ 1, *p* < 0.05) were differentially expressed among the two groups (Figure 3A). In group C9/C0, there were 1758 differentially up-regulated genes and 3247 differentially down-regulated genes (Figure 3A). In group C15/C0, there were 4902 differentially up-regulated genes and 5552 differentially down-regulated genes. Compared with C9/C0, the first phase of viability loss, the expression of a large number of genes was significantly altered under C15/C0, when seed viability had fallen to about 50% (Figure 3A). Meanwhile, in the overlapping region of the two controls, we found 4180 DEGs (Figure 3B). Furthermore, the results of the PCA analysis showed good reproducibility within the groups and clear separation of the three aging comparisons (Figure 3C).

**Figure 4:**
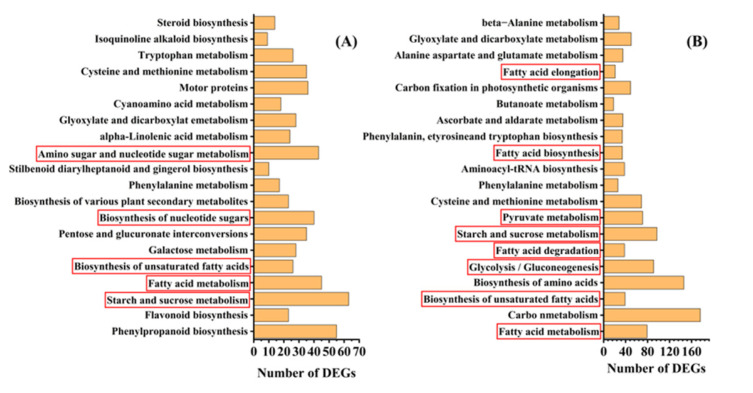
KEGG enrichment analysis of DEGs between (**A**) C9/C0, (**B**) C15/C0 seed aging intervals.

**Figure 5:**
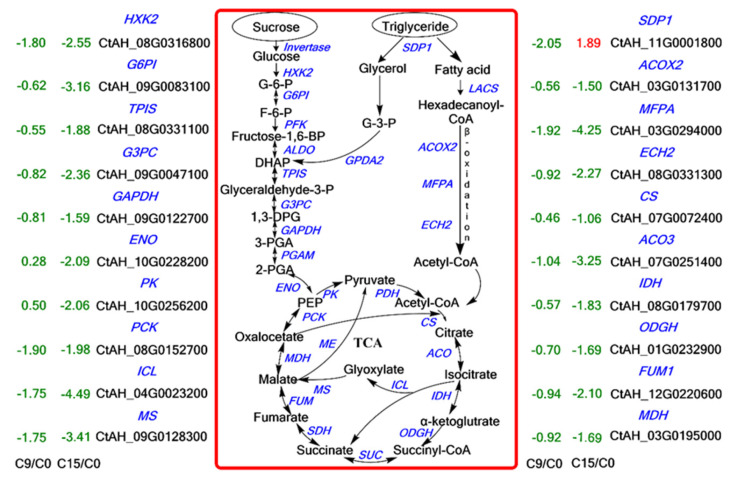
An overview of sugar metabolism, fatty acid degradation, and TCA. The digits in the figure indicate the Log_2_ (FoldChange) values, the red color indicates up-regulated genes, and the green color indicates down-regulated genes. (Enzyme abbreviations are HXK: hexokinase; G6PI: phosphoglycerate isomerase; PFK: 6-phosphofructokinase; ALDO: fructose-bisphosphate aldolase; TPIS: triosephosphate isomerase; GAPDH: glyceraldehyde 3-phosphate dehydrogenase; PGAM: phosphoglycerate mutase; ENO: enolase; PK: pyruvate kinase; PDH: pyruvate dehydrogenase; CS: citrate synthase; ACO: aconitase; IDH: isocitrate dehydrogenase; OGDH: 2-oxoglutarate dehydrogenase; SUC: succinyl-CoA synthase; SDH: succinate dehydrogenase; FUM: fumarase; MDH: malate dehydrogenase. PCK: phosphoenolpyruvate carboxykinase; MS: malate Synthase; ICL: isocitrate lyase; ME: malic enzyme).

To understand the metabolic pathways that are most important during safflower seed aging, we mapped out these processes by functionally annotating DEGs according to the KEGG database. This showed that the up-regulated and down-regulated genes were mainly focused on the cellular metabolism and biosynthesis of energy substances, such as starch, sucrose, and fatty acids (Figure 4). Therefore, we focused on these pathways to decipher the underlying molecular mechanisms of safflower seed aging. KEGG analysis revealed that glycolysis, fatty acid metabolism, and TCA were significantly enriched during seed aging, and we mapped the relevant components in these pathways (Figure 5). The expression of genes related to glycolysis and TCA pathways was almost entirely down-regulated during aging (Figure 5), and the glyoxylate cycle followed the same trend (Figure 5 and Table S1). Regarding fatty acid metabolism, the expression of genes encoding fatty acid biosynthesis and fatty acid degradation were almost entirely down-regulated (Figure 5 and Figure S2). However, the expression of *SDP1* genes that promote TAG catabolism was up-regulated. Furthermore, glycolysis establishes a link with the pentose phosphate pathway (PPP) through *glucose-6-phosphate (G-6-P)*. Important catabolic enzymes of the PPP, including *glucose-6-phosphate dehydrogenase (G6PD)* and *6-phosphogluconolactonase (PGLS)*, consistently show down-regulation of expression (Table S1).

### 2.3. Antioxidant Activity and DNA Damage and Repair Genes during Aging Revealed by KEGG Analysis

During seed aging, excess accumulation of ROS can damage the seeds. Seed antioxidant enzymes play an important role in scavenging ROS to prevent damage. However, we found that the expression patterns for important antioxidant enzymes such as *superoxide dismutase (SOD)*, *peroxidase (POD)*, *CAT*, and *L-ascorbate peroxidase (APX)* were significantly down-regulated during seed aging (Table S1). In addition, genes encoding enzymes involved in the ascorbate–glutathione (AsA–GSH) cycle, including *monodehydrogenase reductase (MDHAR)*, *glutathione S-transferase (GST)*, and *glutathione reductase (GR)*, were also down-regulated during seed aging (Table S1). This result suggests that the capacity of seeds to scavenge ROS decreases during aging, which could potentially lead to an excessive accumulation of ROS and reduction in seed vigor over time.

More importantly, seed viability depends on the stability and integrity of DNA and the ability of seeds to repair DNA damage caused by negative factors [31]. Nucleotides are the basic units that make up DNA and RNA. The genes involved in synthesizing nucleotides were down-regulated (Table S2). Meanwhile, most of the DNA replication and repair genes were also significantly down-regulated (Table S2). In contrast, PCNA, which is associated with DNA replication, DNA repair, chromatin remodeling, cell cycle control [32], and its gene expression was significantly up-regulated with aging time (Table S2). The results indicated that the balance between damage and synthesis and repair of seed genetic material was disrupted during aging, which ultimately led to less stability and integrity of the seed genetic material.

### 2.4. Quantitative Real-Time PCR (qPCR) Verification for Expressions of Aging Altered Genes

**Figure 6:**
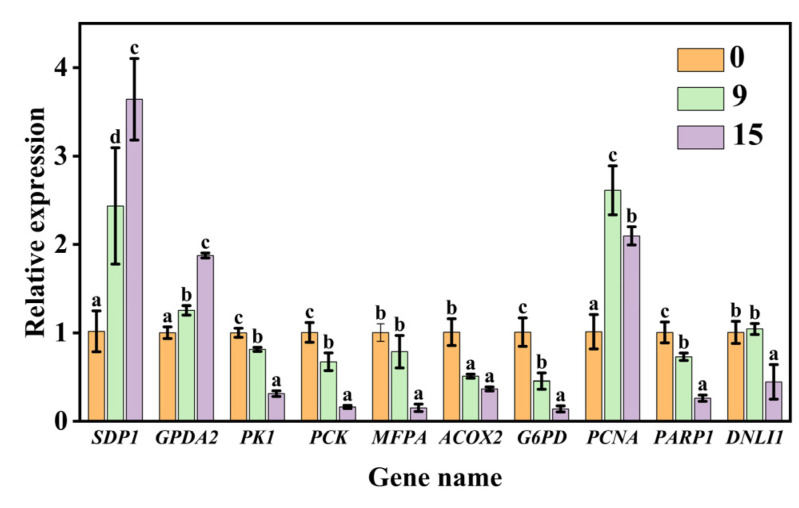
Validation using qPCR. Data are mean ± SE. Different lowercase letters indicate significant differences among aging treatments of 0, 9, and 15 days (*p* < 0.05).

We further validated 10 genes involved in sugar metabolism, fatty acid metabolism, and DNA replication and repair that were significantly altered during aging based on the transcriptome analysis. The results of qPCR analysis showed that CDT aging caused a significant up-regulation in gene expression levels of *SDP1*, *GPDA2*, and *PCNA*. In contrast, there was a significant down-regulation in the gene expression levels of *PK1*, *MFPA*, *ACOX2*, *PCK*, *PARP1*, *G6PD*, and *DNLI1* during aging (Figure 6). The results between qPCR and transcriptome profile for these 10 genes were basically analogous. Overall, these findings indicated the reliability of the transcriptome data for the unraveling of the temporal gene expression patterns during seed aging.

### 2.5. CDT Increases the Level of Certain Fatty Acids and Decreases the Level of Soluble Sugars in Safflower Seeds

**Figure 7:**
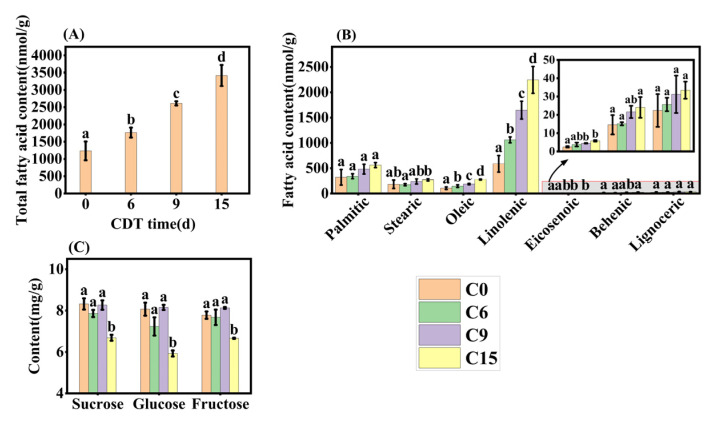
Changes in fatty acid and soluble sugar content under CDT for 6 (C6), 9 (C9), and 15 days (C15). (**A**) Total fatty acid content; (**B**) changes in the content of seven fatty acids; and (**C**) contents of sucrose, glucose, and fructose. Data are mean ± SE. Different lowercase letters indicate significant differences among treatments (*p* < 0.05).

Representative aged seeds were selected based on the results of the germination test and transcription analysis and their fatty acid composition was determined using UPLC-MS. Seven fatty acids were detected, with linoleic acid being the major one (Figure 7B), consistent with the findings of Mehrzad Jafari Barmak [33]. The concentrations of total fatty acids and the seven individual fatty acids significantly increased with CDT time (Figure 7A,B). Thus, total FA levels increased by 42.9%, 111.3%, and 176.4%, respectively, compared with C0 (Figure 7A). Linoleic acid showed the most dramatic increase among the seven fatty acids, reaching 80.5%, 180.5%, and 282.4% higher than C0 seeds (Figure 7B). In C15 seeds, palmitic acid, oleic acid, and stearic increased by 75.0%, 160.8%, and 47.9%, respectively, over C0 seeds (Figure 7B). Whilst eicosenoic acid, behenic acid, and lignoceric acids accounted for less than 4% of the total FAs content, their respective contents increased under CDT.

The changes in seed-soluble sugar concentration during CDT were also determined (Figure 7C). Sucrose, glucose, and fructose contents did not show significant changes before C9, but all decreased significantly at C15 (Figure 7C). By C15, sucrose, fructose, and glucose contents decreased from 8.32 mg·g^−1^, 7.78 mg·g^−1^, and 8.07 mg·g^−1^ to 6.69 mg·g^−1^, 6.66 mg·g^−1^, and 5.93 mg·g^−1^, respectively, compared to the C0 (Figure 7C).

### 2.6. Exogenous Sucrose and DA-6 Enhance the Viability of Aged Seeds

**Figure 8:**
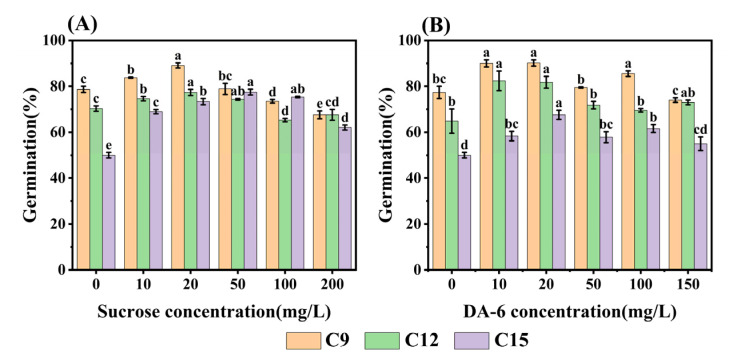
Effects of sucrose and DA-6 on increased safflower seed germination after different degrees of aging up to 15 days (C15). (**A**) Sucrose; and (**B**) DA-6. Data are for seeds seven days after germination. Data are mean ± SE. Different lowercase letters indicate significant differences among treatments (*p* < 0.05).

Based on the results of RNA-seq analysis, it is clear that sugar metabolism and fatty acid metabolism play crucial roles in the germination process of aged safflower seeds. To validate the relationship between energy metabolism and the loss of seed viability, we experimented by adding different concentrations of (exogenous) sucrose and DA-6 during the germination process of aged seeds. It was found that the germination rate of safflower seeds showed an increase and then decreased with the concentration of sucrose (Figure 8A). In C15 seeds, different concentrations of sucrose increased the germination rate of aged safflower seeds, with the highest germination rate (77.48%) observed at a sucrose concentration of 50 mg·L^−1^. Aging of C9 and C12 safflower seeds at sucrose concentrations of 100 and 200 mg·L^−1^ inhibited seed germination, while other concentrations promoted seed germination (Figure 8A). Similarly, DA-6 had a positive effect on aged safflower seeds, but it was not as effective as sucrose for C15 seeds. The germination rate of DA-6 concentration of 50 mg·L^−1^ was only 67% (Figure 8B).

## 3. Discussion

### 3.1. Inactivation of Safflower Seeds Due to Impaired Energy Metabolism

This study showed that a large number of down-regulated genes in energy metabolism were mainly enriched in the pathways of sugar metabolism, fatty acid degradation, and the TCA, which resulted in impaired seed metabolism and energy supply, and ultimately reduced seed germination and vigor. Such observations support the finding that seed nutrient availability and energy supply tend to decrease during storage and lead to significant reductions in germination, germination potential, stem length, and root length of aged seeds [2].

Seed germination and seedling establishment require energy from compounds stored in the seeds. Effective mobilization is then directly and positively correlated with seed vigor and longevity [30]. We observed during safflower seed aging the down-regulation of genes involved in the sugar metabolism pathway, including *HXK2*, *PK*, *ENO*, *GAPDH*, and others (Figure 5). Additionally, *PCK* catalogs the conversion of oxaloacetate to phosphoenolpyruvic acid during gluconeogenesis [34], and its gene expression is also decreased (Figure 5). The main source of energy used by seeds during germination and seedling is from soluble sugars, mainly sucrose, and fructose [35]. Glucose or glycogen is converted to pyruvate or lactate by glycolysis, producing minor levels of ATP under anaerobic or anoxic conditions. The pyruvate then enters the TCA and further produces ATP [36,37]. In Siberian wild rye seeds, inhibition of the sugar-metabolizing enzymes and sugar metabolism-related genes obstructs seed germination and seedling establishment [6]. GAPDH converts glyceraldehyde 3-phosphate to 1, 3-bisphosphoglycerate in the glycolytic pathway, and overexpression of the *GAPDH* gene (OsGAPC3) also elicits a high germination level in rice seeds [38]. Thus, the down-regulation of gene expression for these key enzymes in the glycolysis pathways may impair the energy supply chain during seed aging and affect the germination level.

For oil crops, triacylglycerol is a primary reserve compound in seeds and serves as an energy sucrose [39]. In soybeans, TAGs are hydrolyzed to produce FAs and glycerol, which are then converted to sucrose [9]. During seed aging, we observed a significant up-regulation in the expression of *SDP1* and *GPDA2* genes compared to C0, and an unexpected down-regulation in the expression of *MFPA*, *ACOX2*, *LACS*, *ECH2*, and other genes, which are involved in FAs metabolism (Figure 5 and Figure S2). *SDP1* encodes a platinum-like structural domain-containing triacylglycerol lipase, a key enzyme of TAG hydrolysis to fatty acids and glycerol, which plays a key role in post-emergence growth [13]. Seed FAs are metabolized to acetyl-CoA through β-oxidation or converted to sucrose to provide energy for seed germination [40]. However, in our study, the expression of key enzymes (*ICL* and *MS*) involved in converting FAs to sucrose was down-regulated (Figure 5), suggesting that, as a result of the aging process, FAs could not be rapidly converted to sugar to increase energy for seed germination. This resulted in a large accumulation of FAs, i.e., an increase in concentration, and a decrease in the content of soluble sugars, such as glucose, fructose, and sucrose (Figure 7).

The TCA is the ultimate metabolic pathway for glycolysis and fatty acid catabolism, which enter as acetyl-CoA and are further oxidized to produce ATP [41]. During seed germination, glycolysis, TCA, and PPP (pentose phosphate pathway) are activated [42]. Amino acids produced by proteolysis create alpha-keto acids through transamination, which replenish intermediates of the TCA, such as aspartic acid [43]. Once the glycolysis pathway, PPP, and TCA are activated, most of the stored substances in seeds begin to mobilize to provide material and energy for subsequent seedling growth and development [44]. For example, up-regulation of the expression of *ACO*, *MDH*, and *FUM* encoding aconitase, malate dehydrogenase, and fumarate hydratase, respectively, regulates the synthesis of organic acids and promotes respiratory activity during seed germination, thereby promoting seed germination [45]. However, in our study, the expression of *ACO*, *IDH*, *ODGH*, *SDH*, *FUM*, *MDH*, and *CS* genes was mostly down-regulated during aging (Figure 5), causing a severe reduction in seed energy supply. Among them, FUM, MDH, and ODGH may be targeted for TCA pathway regulation [46]. Furthermore, the PPP is a supplemental source of energy metabolism that directly oxidizes sugar and complements glycolysis to provide cells with NADPH and material to sustain seed germination, as well as providing pentose phosphate for nucleotide metabolism [47], the expression of its key genes was also down-regulated (Table S1). Taken together, it appears that the entire energy supply chain in safflower seeds is severely damaged during the aging process and increasingly fails to provide sufficient energy for seed germination and seedling establishment.

### 3.2. Oxidative Damage and DNA Damage during Safflower Seeds Aging

Seeds that are exposed to CDT produce ROS, MDA, and 4-hydroxynonenal [48]. According to the Free Radical Theory of Ageing, the excessive accumulation of ROS concentrations leads to the deleterious oxidation of critical substances in seed cells, including proteins, DNA, RNA, and cell membrane lipids, and reduces cellular integrity [2]. The accumulation of ROS during seed aging has been well documented [49,50]. ROS will directly attack the deoxyribose carbon to abstract hydrogen, leading to single and double-stranded DNA breaks [51]. MDA, which is a by-product of the peroxidation of unsaturated lipids, is often used as an indicator of seed aging because of its excessive accumulation during seed storage [52]. The increase in MDA with increasing time of aging in this CDT study indicates that the plasma membrane is significantly disrupted (Figure 1D).

High levels of seed vigor and genetic integrity rely on a delicate balance between ROS production and antioxidant capacity [53]. Seeds have antioxidant enzyme systems such as POD, SOD, and CAT, as well as non-enzymatic antioxidant systems such as vitamin E and the AsA–GSH cycle, that help scavenge ROS. Here, we observed a down-regulation in the gene expression of several peroxidases (*SOD*, *POD*, *CAT*, and *APX*) under CDT (Table S1) and a significant decrease in CAT activity (Figure 1E). Several studies have shown that a significant reduction in peroxidase activity is one of the main causes of seed aging in oats [54], Oryza sativa L. [55], and other crops. In plants, the AsA–GSH cycle acts as an antioxidant system involving enzymatic and nonenzymatic antioxidants that play a crucial role in the detoxification of ROS [56]. In oat seeds, artificial aging caused the loss of vigor by the dysfunctioning of GST, a member of the AsA–GSH cycle antioxidant system [57]. Similarly, we found that *MDHAR*, *GR*, and *GST* were significantly down-regulated under CDT (Table S1). This result suggests that the decrease in antioxidants leads to an excessive accumulation of ROS during aging and contributes to decreased seed viability.

As seeds age, the degradation of lipids during seed storage leads to changes in membrane structure and oil particle structure [5], which makes it easier for ROS to damage genome integrity. This damage causes mutations, drift of genes, and loss of genetic integrity, which is a major issue for germplasm conservation over the longer term [58]. In all organisms, genome integrity is maintained by a specific set of DNA repair pathways [59], but in our study, the DNA replication-related gene was down-regulated (Table S2), and the DNA repair pathways were also affected (Table S2). Excessive ROS production leads to the formation of 8-oxo guanine (8-oxo-G), which can induce a C: G to A: T translocation mutation [60]. Although our findings indicate a subtle rebalance between DNA damage and repair during the aging process in safflower seeds, the mechanism still warrants further investigation, including metabolic imbalances. Animal cell studies have shown that FA acid oxidation is involved in DNA double-strand break repair by providing acetyl co-enzyme A to promote the acetylation of PARP1 [61], and PARPs also have regulatory DNA repair properties in the model plant Arabidopsis [62]. The association between material metabolism and genetic material in seed aging also needs to be elucidated.

Previous studies have demonstrated that DA-6 can promote germination and seedling establishment in senescent soybean seeds by encouraging TAG hydrolysis and the conversion of FAs to sucrose [11]. Therefore, we treated aging safflower seeds with exogenous sucrose and DA-6, which resulted in significantly increased germination levels. This finding further supports the important role of impaired sugar and FA metabolism in the aging process of safflower seeds (Figure 8). Moreover, research has shown that CSN improves germination and seedling establishment in aged sunflower seeds by increasing FA conversion into sucrose [12]. Another study found that exogenous spermidine promotes sorghum seed germination by promoting starch and sugar utilization [10]. As safflower is an essential economic crop that relies on high-quality seeds for efficient and effective plant production, the ability to enhance germination in aged seeds potentially has huge implications for agriculture. Such a simple, effective, and affordable method to enhance seed viability may also serve as an excellent model system to investigate underlying mechanisms of aging and recovery.

## 4. Materials and Methods

### 4.1. Plant Material

Seeds of safflower (*Carthamus tinctorius* L.) were collected from Kunming of Yunnan Province, China, in June 2022. The seeds were dried under natural conditions. The original germination level was 98%, the moisture content (MC) was 0.072 g H_2_O·g^−1^ DW, and the thousand kernel weight was 41.638 g. Before the experiments, the seeds were held under medium-term storage conditions of <45% RH and 4 °C in sealed containers.

### 4.2. Controlled Deterioration Treatment (CDT)

The assay of controlled deterioration treatment (CDT) was performed according to the protocol described by Zhou et al. [30]. Seeds were surface sterilized using a 5% sodium hypochlorite solution for 5 min before CDT and then rinsed three times with distilled water, after which seeds were rapidly dried to the original MC. A LiCl solution was made by adding 210 g of anhydrous lithium chloride to 300 mL of distilled water to achieve an environment RH of 60% in the airtight plastic boxes. The seeds were placed in airtight plastic boxes and equilibrated at 20 °C for 24 h, which was recorded as C0, after which the seeds were transferred to a constant temperature aging chamber at 50 °C to continue the aging experiment. Finally, we obtained seeds aged 0, 6, 9, 12, 15, and 18 days (and coded as C0, C6, C9, C12, C15, and C18) and evaluated germination and seedling establishment. Afterwards, to better understand the changes in biological activities during germination of aged safflower seeds, safflower seeds imbibed on 1% agar medium for 12 h were used for physiological and biochemical, transcriptomic, and qPCR analysis. Differences in the application of the analyses at each stage of aging are specified in the Results.

### 4.3. Germination Test

After the seeds were sterilized by immersion in 5% sodium hypochlorite solution as described above, seeds were incubated on 1% agar-water medium at 25 °C under dark conditions. Twenty-five seeds were placed in each Petri dish and four replicates for each germination assessment. Germination was recorded after seven days with germination defined as radicle emergence by >2mm. The parameters of germination that were determined were [10]:

Germination rate = the number of normally germinated seeds on the 7th day/the total number of tested seeds × 100%

Germination potential = the number of normally germinated seeds on the 2nd day/the total number of tested seeds × 100%

Germination index = ∑(Gt/Dt)

where Gt is the number of germinations on the 7 days; Dt is the number of germination days.

### 4.4. Seedling Establishment Test

The seedling establishment tests in aged safflower seeds are used according to a published method [30]. For each treatment (C0, C6, C9, C12, C15, C18), 55 seeds were sown uniformly in peat soil in pots, covered with a thin layer of soil after sowing and watered well in three replications. They were grown in a greenhouse RH of 75%, day/night temperatures of 20°/15 °C, and photoperiods of 8 h day/16 h night. Seedling establishment was evaluated after 21 days, and 30 true-leafed seedlings (10 per replicate) were taken from each (aging) level to be scanned and counted using a root scanner (Zhongjing ScanMaker i800plus, Shanghai Zhongjing Technology Co., Ltd, Shanghai, China) on seedling roots. The belowground parts were placed in an oven at 105 °C for 30 min, then placed in an oven at 80 °C and weighed with an analytical balance. Subsequently, the following biomass, root length, and root surface area were quantified.

### 4.5. Content of MDA

MDA content was determined with kits from Solarbio (Beijing, China). Briefly, about 0.1 g of dried seed tissue was weighed, and 1 mL of extract was added and homogenized in an ice bath. After centrifugation at 8000× *g* for 5 min at 4 °C, the supernatant was taken and placed on ice to be measured. The extracted enzyme solution was quickly mixed with thiobarbituric acid solution and reacted in a water bath at 100 °C for exactly 60 min, cooled in an ice bath, and centrifuged at 10,000× *g* for 10 min at room temperature. Finally, the 200 μL supernatant was taken to determine the absorbance at 600 nm and 532 nm to calculate the MDA content (nmol·g^−1^). Biological replicates were performed, with three replicates for each sample.

### 4.6. CAT Activity Quantification

CAT content was determined using kits from Solarbio. In short, about 0.1 g of dried seed tissue was weighed, and 1 mL of extract was added and homogenized in an ice bath. After centrifugation at 8000× *g* for 10 min at 4 °C, the supernatant was taken and placed on ice to be measured. A 10 μL aliquot of supernatant and 190 μL of working solution were mixed immediately, and the initial absorbance at 240 nm and the absorbance after 1 min were recorded to calculate the CAT activity. The catalyzed degradation of 1 μmol of H_2_O_2_ per gram of tissue in the reaction system per minute was defined as one unit of enzyme activity (U·g^−1^). Biological replicates were performed, with three replicates for each sample.

### 4.7. Determination of Chlorophyll Content

For each sample, 0.1 g of leaves from the fresh seedling were clipped to prepare lyophilized powder. After adding 1 mL of 80% acetone and extracting for 24 h at 4 °C in the dark, the supernatant was centrifuged at 16,000× *g* for 10 min, the supernatant was taken and fixed with 6 mL of 80% acetone, after which the absorbance values were determined by a UV-1900 spectrophotometer (AOE Instruments, Shanghai, China) at 663 nm and 645 nm, and the chlorophyll content was calculated using the following formula [63]:

Ca = 12.72 A 663 − 2.59 A 645

Cb = 22.88 A 645 − 4.67 A 663

CT = Ca + Cb = 20.29 A 645 + 8.05 A 663

where Ca is chlorophyll a content (mg·g^−1^), Cb is chlorophyll b content (mg·g^−1^) and CT is total chlorophyll content (mg·g^−1^); A663 and A645 stand for the absorbance of the sample at the wavelengths of 663 nm and 645 nm, respectively.

### 4.8. Transcriptome Profiling

Samples for transcriptome analysis were collected directly after 12 h imbibition of seeds aged for 0, 9, and 15 d. The hard seed coat was removed and the remaining tissue was frozen in liquid nitrogen. RNA was isolated from the homogenized samples and quantified using an Agilent 2100 Bioanalyzer (Santa Clara, CA, USA). The sequencing library was generated using the following steps. The starting total RNA was used to enrich mRNA with polyA tail, followed by random interruption of the obtained mRNA with divalent cations in the Fragmentation Buffer. Then, the first strand of cDNA was synthesized in the M-MuLV reverse transcriptase system using fragmented mRNA as a template and random oligonucleotides as primers. The RNA strand was then degraded using RNaseH, and the second strand of cDNA was synthesized using dNTPs in the DNA polymerase I system. The purified double-stranded cDNA was then end-repaired, A-tailed, and ligated into sequencing junctions. The cDNA was screened with AMPure XP beads for cDNAs of 370–420 bp, amplified by PCR, and the PCR products were purified using AMPure XP beads again to obtain the final library. The sequencing library was then sequenced on the NovaSeq 6000 platform (Illumina, San Diego, CA, USA) by BGI Genomics. Reference genomes and annotation files followed Wu et al. [64].

### 4.9. Real-Time Quantitative PCR

Gene expression was verified using quantitative real-time polymerase chain reaction (qPCR), and 10 DEGs were selected for qPCR analysis. Total RNA was extracted from C0, C9, and C15 safflower seeds using TransZol Up Plus RNA Kit (Transgen, Beijing, China). The total RNA was treated with PrimeScript™ RT reagent Kit with gDNA Eraser (TaKaRa, Kusatsu, Japan) to remove contaminating genomic DNA. The target gene and one housekeeping gene (25s) were designed using NCBI and synthesized by DynaTech Biotech (Chengdu, China), and the primer sequences are listed in Table S3. An aliquot of 20 µL of PCR mixture consisting of 10 µL of TB Green Premix Ex Taq II (TliRNaseH Plus) (TaKaRa, Liaoning, China), 1 µL of PCR forward primer, 1 µL of PCR reverse primer, 1 µL of DNA template and RNase-free water in a total volume of 20 µL. The annealing temperature varied for different genes. The 2^−∆∆CT^ method was used to calculate the relative expression of circular RNA [65].

### 4.10. Fatty Acids Extraction and Measurements

Safflower seeds (C0, C6, C9, and C15) that had been shelled were ready for extraction of fatty acids as a lyophilized powder. Briefly, about 15 mg of lyophilized powder was taken from each sample and 80 mL (10 mg·mL^−1^) of internal standard was added. In addition, 2 mL of dichloromethane and 2 mL of methanol were added and the mixture was rotated for 1 h. The mixture was left overnight for the proteins to precipitate. Then, 2 mL of dichloromethane and 1.6 mL of ultrapure water were added and vortex centrifuged. The lower clarified liquid was removed, and 4 mL of dichloromethane was added to extract the remaining upper phase and repeated twice. The lower liquid fraction was mixed, blown dry with nitrogen, redissolved in 1 mL isopropanol, and passed through a 0.22 μm organic filter membrane prior to testing.

Analyses were performed using an ultra-performance liquid chromatography (UPLC) system (Shimadzu, Kyoto, Japan) and a triple quadrupole tandem mass spectrometer (Triple TOF, AB Sciex, Framingham, MA, USA). All samples were quantitatively analyzed by high-resolution LC-MS for positive and negative modes and structurally analyzed by LC-MS/MS for positive modes. Analytical conditions were as follows: chromatographic system, shimadzu UPLC LC-30A (Shimadzu, Kyoto, Japan); column, Phenomenex Kinete C18 column, 100 × 2.1 mm, 2.6 µm in diameter (Phenomenex, Torrance, CA, USA); injection volume, 3 μL; flow rate, 0.4 mL·min^−1^; column temperature, 60 °C; sample compartment temperature, 4 °C; A-phase, H_2_O: MeOH: ACN (1:1:1 with 5 mM NH4Ac); B-phase, IPA: CAN (5:1) (with 5 mM NH_4_Ac); gradient elution conditions: 0.5 min, 20% B phase; 1.5 min, 40% B phase; 3 min, 60% B phase; 13 min, 98% B phase; 13.1, min 20% B phase; 17 min, 20% B phase. Mass spectrometry system, AB Sciex TripleTOF^®^ 6600 (Triple TOF, AB Sciex, Framingham, MA, USA), ESI ion source, positive mode, the mass number range of the mass spectrometry acquisition was *m*/*z* 100–1200, the mass spectrometry conditions were as follows: curtain Gas, 35.000 psi; ion Source Gas1, 50.000; ion Source Gas2, 50.00; ionSpray Voltage, 5500.00 V; Temperature, 600 °C.

### 4.11. Determination of Various Sugar Content

The sugar content (mg·g^−1^) in safflower seeds was determined according to a published method [66]. Safflower seeds (C0, C6, C9, and C15) that had been shelled were ready for extraction of soluble sugar as a lyophilized powder. Samples of 200 mg were put into a 10 mL scaled centrifuge tube with the addition of 5 mL of 80% ethanol solution placed in an 80 °C water bath for 10 min and shaken three to five times in the process. The supernatant was collected by centrifugation at 5000× *g* for 5 min. The above operation was repeated and the supernatants were combined. The extracts were filtered through a 0.22 μm organic phase filter. Sugar content was determined by direct HPLC measurements using a water binary HPLC system equipped with a refractive index detector. Analytical conditions were as follows: column Agilent Hi-Plea Ca (8% cross-linked), 300 × 7.7 mm, 8 µm in diameter (Agilent Technologies, Inc., Santa Clara, CA, USA); column temperature, 85 °C; mobile phase, Milli-Q water; and flow rate, 0.6 mL·min^−1^. Data were collected and processed by the Waters chromatography station DataApex (Waters, Milford, USA). Sugars were identified by comparison with retention times and coelution of authentic standard solutions.

### 4.12. Exogenous Sucrose and DA-6 Treatment of Aged Safflower Seeds

Seeds aged for 9, 12, and 15 days (C9, C12, and C15) were sterilized by immersion in 5% sodium hypochlorite solution as previously described. The seeds were then incubated on 1% agar medium containing sucrose at concentrations of 0, 10, 20, 50, 100, 200 mg·L^−1^ and DA-6 at concentrations of 0, 10, 20, 50, 100, 150 mg·L^−1^ at 25 °C under dark conditions. Twenty-five seeds were placed in each Petri dish and four replicates for each germination assessment. Germination was recorded after seven days with germination defined as radicle emergence by >2mm.

### 4.13. Statistical Analysis

One-way analysis of variance (ANOVA) was used to compare differences between treatments using SPSS 26.0 with Duncan’s multiple range method, and the significance level was set at *p* < 0.05. The values are expressed as mean ± SE.

## 5. Conclusions

**Figure 9:**
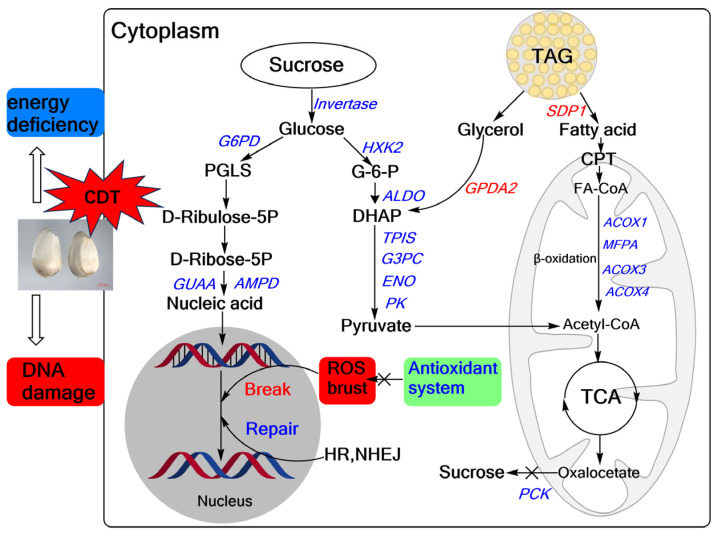
Overview of safflower seed differential genes during CDT. Reactive oxygen species, ROS; glycolysis, fatty acid metabolism, tricarboxylic acid cycle (TCA), pentose phosphate pathway (PPP), antioxidant system, DNA repair (HR, NEHJ). The red text represents upward adjustments and the blue labeling represents downward adjustments during CDT.

In summary, we propose a model for CDT-mediated seed aging in safflower (Figure 9). Our study found that CDT decreased the antioxidant capacity of safflower seeds, increased cell membrane permeability, and significantly reduced seed viability and seedling growth. Meanwhile, transcriptomic analysis revealed CDT-induced changes in genes involved in sugar metabolism, fatty acid metabolism, and DNA repair. Specifically, fatty acids generated from TAG catabolism by seeds during aging are not efficiently converted to sucrose, which in turn leads to energy deficits. At the same time, the reduced capacity for nucleotide synthesis and the deterioration of the DNA repair ability exacerbate DNA damage, leading to the loss of safflower seed vigor. These findings provide essential information on the molecular mechanisms that influence safflower seed aging. However, elucidating the complex regulatory network among these identified DEGs and their functions in aging is challenging, especially the relationship between cell metabolism and changes in genetic material.

## Data Availability

All data supporting the findings of this study are available within the paper and within its Supplementary Data, which are published online.

## Supplementary Materials

The following supporting information can be downloaded at: https://www.mdpi.com/article/10.3390/plants13050659/s1, Table S1: The detailed information on the glyoxylate cycle and pentose phosphate pathway genes; Table S2: Log_2_ (Fold changes) of DEGs in nucleotide synthesis, DNA replication, and DNA repair pathway; Table S3: gene primers of qPCR.; Figure S1: Seedlings from C0, C6, C9, C12, C15, and C18 (days) aged seeds; Figure S2: the expression pattern of the genes related to fatty acid-related KEGG terms.

## References

[1] Reed R.C., Bradford K.J., Khanday I. (2022). Seed germination and vigor: Ensuring crop sustainability in a changing climate. Heredity.

[2] Zhou W., Chen F., Luo X., Dai Y., Yang Y., Zheng C., Yang W., Shu K. (2020). A matter of life and death: Molecular, physiological, and environmental regulation of seed longevity. Plant Cell Environ..

[3] Shu Y., Zhou Y., Mu K., Hu H., Chen M., He Q., Huang S., Ma H., Yu X. (2020). A transcriptomic analysis reveals soybean seed pre-harvest deterioration resistance pathways under high temperature and humidity stress. Genome.

[4] Wang L., Ma H., Song L., Shu Y., Gu W. (2012). Comparative proteomics analysis reveals the mechanism of pre-harvest seed deterioration of soybean under high temperature and humidity stress. J. Proteom..

[5] Chen C., Wang R., Dong S., Wang J., Ren C.X., Chen C.P., Yan J., Zhou T., Wu Q.H., Pei J. (2022). Integrated proteome and lipidome analysis of naturally aged safflower seeds varying in vitality. Plant Biol..

[6] Lei X., Liu W., Zhao J., You M., Xiong C., Xiong Y., Xiong Y., Yu Q., Bai S., Ma X. (2020). Comparative Physiological and Proteomic Analysis Reveals Different Involvement of Proteins during Artificial Aging of Siberian Wildrye Seeds. Plants.

[7] Xiong Y., Ren Y., Li W., Wu F., Yang W., Huang X., Yao J. (2019). NF-YC12 is a key multi-functional regulator of accumulation of seed storage substances in rice. J. Exp. Bot..

[8] Goepfert S., Poirier Y. (2007). β-Oxidation in fatty acid degradation and beyond. Curr. Opin. Plant Biol..

[9] Theodoulou F.L., Eastmond P.J. (2012). Seed storage oil catabolism: A story of give and take. Curr. Opin. Plant Biol..

[10] Zhang M., Li B., Wan Z., Chen X., Liu C., Liu C., Zhou Y. (2022). Exogenous Spermidine Promotes Germination of Aged Sorghum Seeds by Mediating Sugar Metabolism. Plants.

[11] Zhou W., Chen F., Zhao S., Yang C., Meng Y., Shuai H., Luo X., Dai Y., Yin H., Du J. (2019). DA-6 promotes germination and seedling establishment from aged soybean seeds by mediating fatty acid metabolism and glycometabolism. J. Exp. Bot..

[12] Huang Y., Cai S., Ruan X., Xu J., Cao D. (2021). CSN improves seed vigor of aged sunflower seeds by regulating the fatty acid, glycometabolism, and abscisic acid metabolism. J. Adv. Res..

[13] Eastmond P.J. (2006). *SUGAR-DEPENDENT1* encodes a patatin domain triacylglycerol lipase that initiates storage oil breakdown in germinating Arabidopsis seeds. Plant Cell.

[14] Quettier A.L., Shaw E., Eastmond P.J. (2008). SUGAR-DEPENDENT6 encodes a mitochondrial flavin adenine dinucleotide-dependent glycerol-3-p dehydrogenase, which is required for glycerol catabolism and post germinative seedling growth in Arabidopsis. Plant Physiol..

[15] Manova V., Gruszka D. (2015). DNA damage and repair in plants–from models to crops. Front. Plant Sci..

[16] Que Q., Chen Z., Kelliher T., Skibbe D., Dong S., Chilton M.D. (2019). Plant DNA Repair Pathways and Their Applications in Genome Engineering. Methods Mol. Biol..

[17] Dantas A.F., Fascineli M.L., José S.C.B.R., Pádua J.G., Gimenes M.A., Grisolia C.K. (2019). Loss of genetic integrity in artificially aged seed lots of rice (*Oryza sativa* L.) and common bean (*Phaseolus vulgaris* L.). Mutat. Res. Genet. Toxicol. Environ. Mutagen..

[18] Waterworth W.M., Bray C.M., West C.E. (2015). The importance of safeguarding genome integrity in germination and seed longevity. J. Exp. Bot..

[19] Nisa M., Bergis C., Pedroza-Garcia J.A., Drouin-Wahbi J., Mazubert C., Bergounioux C., Benhamed M., Raynaud C. (2021). The plant DNA polymerase theta is essential for the repair of replication-associated DNA damage. Plant J..

[20] Ray A., Langer M. (2002). Homologous recombination: Ends as the means. Trends Plant Sci..

[21] Fell V.L., Schild-Poulter C. (2012). Ku regulates signaling to DNA damage response pathways through the Ku70 von Willebrand A domain. Mol. Cell Biol..

[22] Cadet J., Wagner J.R. (2013). DNA base damage by reactive oxygen species, oxidizing agents, and UV radiation. Cold Spring Harb. Perspect. Biol..

[23] Margaret B.F., Christopher M.R., Christina W. (2017). Decline in RNA integrity of dry-stored soybean seeds correlates with loss of germination potential. J. Exp. Bot..

[24] Ekin Z. (2005). Resurgence of Safflower (*Carthamus tinctorius* L.) Utilization: A Global View 2002. J. Agron..

[25] Xin L., Guo L., Edirs S., Zhang Z., Cai C., Yang Y., Lian Y., Yang H. (2022). An Efficient Deacidification Process for Safflower Seed Oil with High Nutritional Property through Optimized Ultrasonic-Assisted Technology. Molecules.

[26] Chakradhari S., Perkons I., Mišina I., Sipeniece E., Radziejewska-Kubzdela E., Grygier A., Rudzińska M., Patel K.S., Radzimirska-Graczyk M., Górna’s P. (2020). Profiling of the bioactive components of safflower seeds and seed oil: Cultivated (*Carthamus tinctorius* L.) vs. wild (*Carthamus oxyacantha* M. Bieb.). Eur. Food Res. Technol..

[27] Pukacka S., Kuiper P.J.C. (1988). Phospholipid composition and fatty acid peroxidation during ageing of *Acer platanoides* seeds. Physiol. Plant..

[28] Goel A., Sheoran I.S. (2003). Lipid peroxidation and peroxide-scavenging enzymes in cotton seeds. Biol. Plant..

[29] Kumari A. (2009). Germination behaviour, viability and longevity of safflower (*Carthamus tinctorius* L.) seeds. Biosciences.

[30] Zhou L., Lu L., Chen C., Zhou T., Wu Q., Wen F., Chen J., Pritchard H.W., Peng C., Pei J. (2022). Comparative changes in sugars and lipids show evidence of a critical node for regeneration in safflower seeds during aging. Front. Plant Sci..

[31] Kiran K.R., Deepika V.B., Swathy P.S., Prasad K., Kabekkodu S.P., Murali T.S., Satyamoorthy K., Muthusamy A. (2020). ROS-dependent DNA damage and repair during germination of NaCl primed seeds. J. Photochem. Photobiol. B.

[32] Strzalka W., Ziemienowicz A. (2011). Proliferating cell nuclear antigen (PCNA): A key factor in DNA replication and cell cycle regulation. Ann. Bot..

[33] Barmak M.J., Nouri E., Shahraki M.H., Ghalamfarsa G., Zibara K., Delaviz H., Ghanbari A. (2023). Safflower seed oil, a rich source of linoleic acid, stimulates hypothalamic neurogenesis in vivo. Anat. Cell Biol..

[34] Zeng W., Peng Y., Zhao X., Wu B., Chen F., Ren B., Zhuang Z., Gao Q., Ding Y. (2019). Comparative Proteomics Analysis of the Seedling Root Response of Drought-sensitive and Drought-tolerant Maize Varieties to Drought Stress. Int. J. Mol. Sci..

[35] Graham I.A. (2008). Seed storage oil mobilization. Annu. Rev. Plant Biol..

[36] Plaxton W.C. (1996). The organization and regulation of plant glycolysis. Annu. Rev. Plant Physiol. Plant Mol. Biol..

[37] Chang L., Ni J., Beretov J., Wasinger V.C., Hao J., Bucci J., Malouf D., Gillatt D., Graham P.H., Li Y. (2017). Identification of protein biomarkers and signaling pathways associated with prostate cancer radioresistance using label-free LC-MS/MS proteomic approach. Sci. Rep..

[38] Tan L., Chen S., Wang T., Dai S. (2013). Proteomic insights into seed germination in response to environmental factors. Proteomics.

[39] Kelly A.A., Quettier A.L., Shaw E., Eastmond P.J. (2011). Seed storage oil mobilization is important but not essential for germination or seedling establishment in Arabidopsis. Plant Physiol..

[40] Kindl H. (1993). Fatty acid degradation in plant peroxisomes: Function and biosynthesis of the enzymes involved. Biochimie.

[41] Zhu M., Zang Y., Zhang X., Shang S., Xue S., Chen J., Tang X. (2023). Insights into the regulation of energy metabolism during the seed-to-seedling transition in marine angiosperm *Zostera marina* L.: Integrated metabolomic and transcriptomic analysis. Front. Plant Sci..

[42] Zaynab M., Kanwal S., Furqan M., Islam W., Noman A., Ali G.M., Rehman N., Zafar S., Sughra K., Jahanzab M. (2017). Proteomic approach to address low seed germination in *Cyclobalnopsis gilva*. Biotechnol. Lett..

[43] Gao W., He Y., Zhang F., Zhao F., Huang C., Zhang Y., Zhao Q., Wang S., Yang C. (2019). Metabolic engineering of *Bacillus amyloliquefaciens* LL3 for enhanced poly-γ-glutamic acid synthesis. Microb. Biotechnol..

[44] Song Y., Gao X., Wu Y. (2021). Key Metabolite Differences Between Korean Pine (*Pinus koraiensis*) Seeds with Primary Physiological Dormancy and No-Dormancy. Front. Plant Sci..

[45] Liu Y., Qu J., Zhang L., Xu X., Wei G., Zhao Z., Ren M., Cao M. (2019). Identification and characterization of the TCA cycle genes in maize. BMC Plant Biol..

[46] Zhang Y., Fernie A.R. (2018). On the role of the tricarboxylic acid cycle in plant productivity. J. Integr. Plant Biol..

[47] Wang S., Shen Y., Bao H. (2021). Morphological, physiological and biochemical changes in *Magnolia zenii* Cheng seed during development. Physiol. Plant.

[48] Wiebach J., Nagel M., Börner A., Altmann T., Riewe D. (2020). Age-dependent loss of seed viability is associated with increased lipid oxidation and hydrolysis. Plant Cell Environ..

[49] Petla B.P., Kamble N.U., Kumar M., Verma P., Ghosh S., Singh A., Rao V., Salvi P., Kaur H., Saxena S.C. (2016). Rice PROTEIN l-ISOASPARTYL METHYLTRANSFERASE isoforms differentially accumulate during seed maturation to restrict deleterious isoAsp and reactive oxygen species accumulation and are implicated in seed vigor and longevity. New Phytol..

[50] Ebone L.A., Caverzan A., Chavarria G. (2019). Physiologic alterations in orthodox seeds due to deterioration processes. Plant Physiol. Biochem..

[51] Balasubramanian B., Pogozelski W.K., Tullius T.D. (1998). DNA strand breaking by the hydroxyl radical is governed by the accessible surface areas of the hydrogen atoms of the DNA backbone. Proc. Natl. Acad. Sci. USA.

[52] Liu J., Wang Q., Karagić Đ., Liu X., Cui J., Gui J., Gu M., Gao W. (2016). Effects of ultrasonication on increased germination and improved seedling growth of aged grass seeds of tall fescue and Russian wildrye. Sci. Rep..

[53] Bailly C., El-Maarouf-Bouteau H., Corbineau F. (2008). From intracellular signaling networks to cell death: The dual role of reactive oxygen species in seed physiology. Comptes Rendus Biol..

[54] Kong L., Huo H., Mao P. (2015). Antioxidant response and related gene expression in aged oat seed. Front. Plant Sci..

[55] Gao J., Fu H., Zhou X., Chen Z., Luo Y., Cui B., Chen G., Liu J. (2016). Comparative proteomic analysis of seed embryo proteins associated with seed storability in rice (*Oryza sativa* L.) during natural aging. Plant Physiol. Biochem..

[56] Hasanuzzaman M., Bhuyan M.H.M.B., Anee T.I., Parvin K., Nahar K., Mahmud J.A., Fujita M. (2019). Regulation of Ascorbate-Glutathione Pathway in Mitigating Oxidative Damage in Plants under Abiotic Stress. Antioxidants.

[57] Cheng H., Ma X., Jia S., Li M., Mao P. (2020). Transcriptomic analysis reveals the changes of energy production and AsA-GSH cycle in oat embryos during seed ageing. Plant Physiol. Biochem..

[58] Colville L., Pritchard H.W. (2019). Seed life span and food security. New Phytol..

[59] Waterworth W.M., Masnavi G., Bhardwaj R.M., Jiang Q., Bray C.M., West C.E. (2010). A plant DNA ligase is an important determinant of seed longevity. Plant J..

[60] Van Loon B., Markkanen E., Hübscher U. (2010). Oxygen as a friend and enemy: How to combat the mutational potential of 8-oxo-guanine. DNA Repair.

[61] Yang S., Hwang S., Kim B., Shin S., Kim M., Jeong S.M. (2023). Fatty acid oxidation facilitates DNA double-strand break repair by promoting PARP1 acetylation. Cell Death Dis..

[62] Gu Z., Pan W., Chen W., Lian Q., Wu Q., Lv Z., Cheng X., Ge X. (2019). New perspectives on the plant PARP family: Arabidopsis PARP3 is inactive, and PARP1 exhibits predominant poly (ADP-ribose) polymerase activity in response to DNA damage. BMC Plant Biol..

[63] Makarska-Bialokoz M., Kaczor A.A. (2014). Computational analysis of chlorophyll structure and UV-Vis spectra: A student research project on the spectroscopy of natural complexes. Spectrosc. Lett..

[64] Wu Z., Liu H., Zhan W., Yu Z., Qin E., Liu S., Yang T., Xiang N., Kudrna D., Chen Y. (2021). The chromosome-scale reference genome of safflower (*Carthamus tinctorius* L.) provides insights into linoleic acid and flavonoid biosynthesis. Plant Biotechnol. J..

[65] Livak K.J., Schmittgen T.D. (2001). Analysis of relative gene expression data using real-time quantitative PCR and the 2− ΔΔCT method. Methods.

[66] Zhou T., Qiu X., Zhao L., Yang W.J., Wen F.Y., Wu Q.H., Yan J., Xu B.J., Chen J., Ma Y.T. (2022). Optimal light intensity and quality increased the saffron daughter corm yield by inhibiting the degradation of reserves in mother corms during the reproductive stage. Ind. Crops Prod..

