# Supplementary material for: Aging-Induced Reduction in Safflower Seed Germination via Impaired Energy Metabolism and Genetic Integrity Is Partially Restored by Sucrose and DA-6 Treatment
Source: Plants (Basel). 2024 Feb 27;13(5):659. doi: 10.3390/plants13050659 (PMC10934749; doi:10.3390/plants13050659)
Supplement: Supplementary file 1 [file plants-13-00659-s001.zip › plants-2860885-supplementary.pdf]

Table S1. The detailed information on the glyoxylate cycle and pentose phosphate pathway genes in different seed aging comparison groups (C9/C0 and C15/C0).

Pathway	Gene_id	Gene Name	Log2(Fold Change)	
			C9/C0	C15/C0
glyoxylate cycle	CtAH04G0023200	isocitrate lyase	-1.750	-4.487
	CtAH09G0128300	Malate synthase	-1.746	-3.415
	CtAH09G0017300	NADP-dependent malic enzyme	-2.198	-5.001
	CtAH06G0258700	NADP-dependent malic enzyme	-0.849	-2.635
	CtAH12G0110400	NADP-dependent malic enzyme	-0.209	-1.181
pentose phosphate pathway(PPP)	CtAH07G0159000	6-phosphogluconolactonase	-0.565	-1.076
	CtAH01G0004900	glucose-6-phosphate 1-dehydrogenase	-1.328	-2.169
	CtAH09G0083100	glucose-6-phosphate isomerase	-0.623	-3.164
	CtAH07G0043400	Probable ribose-5-phosphate isomerase 2	0.743	3.061
	CtAH05G0261000	Probable 6-phosphogluconolactonase 4,	-0.734	-0.656
	CtAH07G0158000	Probable 6-phosphogluconolactonase 4	-0.316	-0.646
	CtAH05G0206300	6-phosphogluconolactonase 3	0.249	-0.119
antioxidant activities	CtAH07G0085000	Catalase	-0.447	-1.439
	CtAH11G0124300	Superoxide dismutase	-1.171	-1.799
	CtAH12G0058800	Peroxidase N1	-1.754	-3.834
	CtAH12G0172100	L-ascorbate peroxidase	-0.762	-1.418
	CtAH02G0110700	L-ascorbate peroxidase 3	-0.766	-1.651
	CtAH06G0073800	glutathione reductase	0.459	-1.018
	CtAH03G0274300	Monodehydroascorbate reductase	-0.736	-1.740
	CtAH05G0105600	Probable glutathione S-transferase	-1.196	-1.818

Table S2. Fold changes of DEPs in nucleotide synthesis, DNA replication, and DNA repair pathway in different seed aging comparison groups (C9/C0 and C15/C0).

Pathway	Gene id	Gene Name	Log2(Fold Change)	
			C9/C0	C15/C0
nucleotide synthesis	CtAH01G0101400	Probable GMP synthase	-2.286	-3.373
	CtAH02G0121800	AMP deaminase	-0.169	-1.690
	CtAH10G0100300	CTP synthase	-0.858	-2.044
	CtAH08G0212300	CTP synthase 1-B	0.440	0.322
	CtAH01G0101900	CTP synthase	-0.177	-1.748
	CtAH05G0103100	Bifunctional dihydrofolate reductase-thymidylate synthase 1	0.536	0.026
	CtAH07G0220100	Bifunctional dihydrofolate reductase-thymidylate synthase	-0.252	-2.027
DNA replication	CtAH09G0144300	DNA polymerase I A	-0.152	-1.292
	CtAH06G0053400	DNA ligase 1	-0.228	-1.426
	CtAH01G0210000	flap endonuclease 1	-0.394	-1.710
	CtAH12G0153500	DNA ligase 4	1.045	0.611
	CtAH11G0070100	DNA ligase 6	0.684	1.254
	CtAH10G0058000	proliferating cell nuclear antigen	1.316	1.961
	CtAH08G0248300	DNA polymerase I	-0.152	-0.710
	CtAH05G0104500	DNA replication licensing factor MCM 4	-2.581	-1.280
	CtAH08G0273000	replication factor C subunit 1	-0.324	-2.129
	CtAH09G0144300	DNA polymerase I A	-0.152	-1.292
Homologous recombination (HR)	CtAH06G0283800	Serine/threonine-protein kinase ATM	-0.258	-2.532
	CtAH08G0346500	Fanconi anemia group J protein homolog	0.399	-1.396
	CtAH07G0245700	double-strand break repair protein MRE11	0.497	-1.094
	CtAH12G0192800	ATP-dependent DNA helicase Q-like 3	-0.206	-0.611
	CtAH08G0231100	DNA topoisomerase 3-beta	-0.086	-1.753
nonhomologous end joining (cNHEJ)	CtAH02G0055800	DNA helicase 2 subunit KU70	0.062	-0.794
	CtAH03G0233000	DNA helicase 2 subunit KU80	0.617	-0.720
Others DNA repair pathways	CtAH01G0183700	DNA damage-binding protein 1	-0.018	-1.688
	CtAH10G0060800	DNA damage-binding protein 2	1.511	1.776
	CtAH10G0041700	DNA-3-methyladenine glycosylase	0.176	-0.705
	CtAH10G0038800	DNA polymerase delta small subunit	-0.145	-1.555
	CtAH07G0144800	DNA mismatch repair protein MSH3	0.820	1.414
	CtAH04G0085400	Exonuclease 1	0.512	1.077
	CtAH09G0237400	Poly [ADP-ribose] polymerase 1	-0.937	-2.607

Table S3. gene primers of qPCR.

Number	Gene ID	Primer	For（5’-3’）	Length(bp)
1	CtAH06G0067600	PK1F	ACACCATCTTGTGTGCCGAT	20
2	CtAH06G0067600	PK1R	ACGAGCAACCATGAACGAGT	20
3	CtAH08G0152700	PCK1F	ATACTCGTGCAGCATACCCG	20
4	CtAH08G0152700	PCK1R	ACAAGCCATCCAGTAGCACC	20
5	CtAH10G0123800	SDP1F	AAGAATGGGCTCATGCTGCT	20
6	CtAH10G0123800	SDP1R	CAGATAATGGATCCGGCGCT	20
7	CtAH03G0131700	ACOX2F	ACAAAACACTTCCGGGGGTT	20
8	CtAH03G0131700	ACOX2R	GGCTTCTTCGGAGGACCAAA	20
9	CtAH03G0294000	MFPAF	CCGATGGGGTGGCTATCATC	20
10	CtAH03G0294000	MFPAR	GTCGACATCGCTTTGCCATC	20
11	CtAH09G0237400	PARP1F	AGCGAGTGAAGAGGCGAAAA	20
12	CtAH09G0237400	PARP1R	CCCACTTCCGGAACACATGA	20
13	CtAH12G0162000	PCNAF	ACGCCAATTTCGATTGCTCG	20
14	CtAH12G0162000	PCNAR	CCATCATCGGCCTTGAGAGT	20
15	CtAH01G0004900	G6PDF	GCCACTATGGAACCGTGACA	20
16	CtAH01G0004900	G6PDR	ACCATAGTTGCGAAGGTGGG	20
17	CtAH06G0053400	DNLI1F	GATGCACTCATGTCCAACGC	20
18	CtAH06G0053400	DNLI1R	AGCAAATCATCGGGTGTCGT	20
19	CtAH01G0004900	GPDA2F	TCTCGTTCATGGCGGAACAA	20
20	CtAH01G0004900	GPDA2R	CTGGTACAGCATGGAGGCAA	20

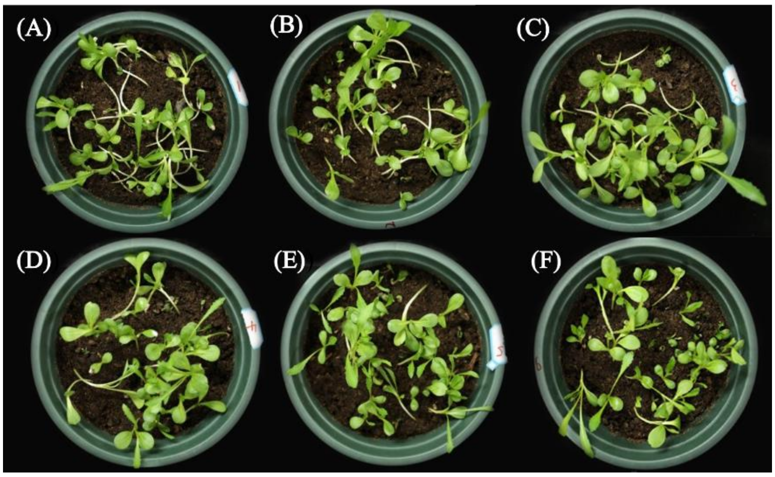

Figure S1. CDT of safflower seeds reduces their seeding establishment. (A-F) Seedlings from C0, C6, C9, C12, C15, and C18 (days) aged seeds.

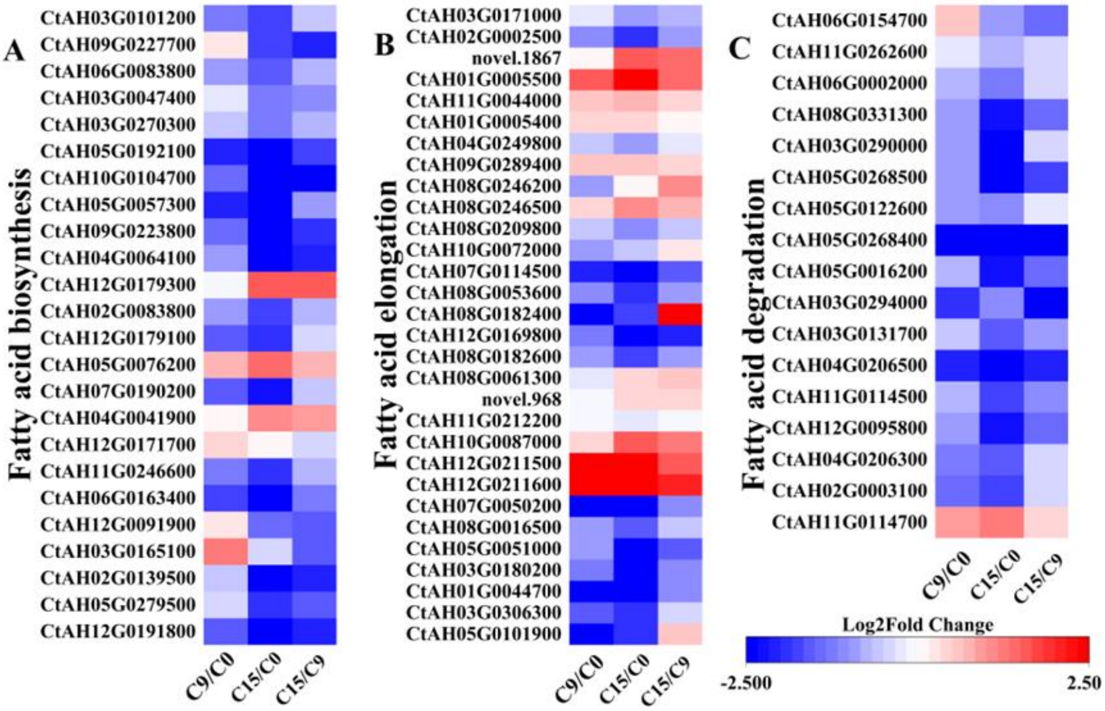

Figure S2. The expression pattern of the genes related to fatty acid-related KEGG terms. Red indicates up-regulated and blue indicates down-regulated.
